# Validation of Fractional Anisotropy and Apparent Diffusion Coefficient Obtained From Diffusion Tensor Imaging With Reverse Encoding Distortion Correction

**DOI:** 10.7759/cureus.97540

**Published:** 2025-11-23

**Authors:** Kazushige Ichikawa, Toshiaki Taoka, Hirohito Kan, Nobuyasu Ichinose, Masanori Ozaki, Akifumi Kamiunten, Shinji Naganawa

**Affiliations:** 1 Department of Radiological Technology, Nagoya University Hospital, Nagoya, JPN; 2 Department of Innovative Biomedical Visualization, Nagoya University Graduate School of Medicine, Nagoya, JPN; 3 Department of Integrated Health Sciences, Nagoya University Graduate School of Medicine, Nagoya, JPN; 4 MRI Systems Division, Canon Medical Systems Corporation, Otawara, JPN; 5 Department of Radiology, Nagoya University Graduate School of Medicine, Nagoya, JPN

**Keywords:** apparent diffusion coefficient, diffusion tensor imaging, fractional anisotropy, magnetic resonance imaging, reverse encoding distortion correction

## Abstract

Background

Diffusion-weighted imaging acquired using echo-planar imaging is susceptible to distortion due to its sensitivity to static magnetic field inhomogeneity and eddy currents induced by motion-probing gradients. Although "topup" and "eddy" in the FMRIB (Functional Magnetic Resonance Imaging of the Brain) Software Library (FSL) are widely used for distortion correction in research, their reliance on offline post-processing, as well as the considerable time and multiple steps required, limits their feasibility in routine clinical practice. Diffusion tensor imaging (DTI) with reverse encoding distortion correction (RDC), which can be obtained directly on the MRI scanner console, reduces post-processing time by eliminating the need for FSL.

Objective

This study aimed to visually assess distortions in non-RDC fractional anisotropy (FA_non-RDC_), topup- and eddy-corrected fractional anisotropy (FA) in FSL (FA_FSL_), and FA_RDC_ relative to MPRAGE (Magnetization-Prepared Rapid Gradient-Echo), and to validate FA and apparent diffusion coefficient (ADC) derived from RDC DTI.

Materials and methods

This prospective study included 10 healthy volunteers (mean age, 40.1 ± 5.7 years). Non-RDC b_0_ images, non-RDC DTI, RDC DTI, and MPRAGE were acquired on a 3T MRI scanner. Non-RDC b_0_ images and non-RDC DTI were subsequently processed using topup and eddy in FSL for distortion correction. FA and ADC were registered to the standard space, and the mean values were calculated for each of the 50 regions of interest (ROIs) defined using the Johns Hopkins University-International Consortium of Brain Mapping (JHU-ICBM)-labels 1 mm white matter atlas. All statistical analyses were performed using Bonferroni-corrected Wilcoxon signed-rank tests, with the significance level set at P < 0.05.

Results

The processing time for RDC DTI was approximately two minutes, whereas topup and eddy in FSL required about 90 minutes per subject. Distortions observed at the cranial base and near the frontal sinuses on FA_non-RDC_ were substantially mitigated in FA_RDC_, comparable to FA_FSL_. Although 10 ROIs in FA_non-RDC_ and nine ROIs in FA_FSL_ showed significant differences compared with FA_RDC_, the FA differences for each ROI were minimal, with a mean ± 95% confidence interval (CI) of −0.003 ± 0.006 and −0.002 ± 0.005. Furthermore, two ROIs in ADC_non-RDC_ and none in ADC_FSL_ showed significant differences compared with ADC_RD__C_; however, the ADC differences for each ROI were minimal, with a mean ± 95% CI of 2 ± 16 and −1 ± 10 × 10^-6^ mm^2^/s.

Conclusion

RDC DTI effectively reduced distortions while yielding FA and ADC values comparable to those of non-RDC DTI and FSL DTI. Given the shorter post-processing time and fewer processing steps, RDC DTI may be considered promising for clinical applications.

## Introduction

Fractional anisotropy (FA) and apparent diffusion coefficient (ADC) derived from diffusion tensor imaging (DTI) are widely used to assess white matter integrity and characterize microstructural tissue properties. In multiple sclerosis, reductions in FA and increases in ADC within normal-appearing white matter and lesions reflect demyelination and axonal degeneration, providing valuable biomarkers for disease monitoring [1]. Alterations in FA and ADC can reveal white matter changes even in mild traumatic brain injury that may be missed by conventional CT or MRI, highlighting their potential as sensitive biomarkers [2]. Beyond neurological applications, FA and ADC have also been investigated for differentiating between benign and malignant breast lesions and for predicting cellularity [3]. Therefore, accurate measurement of these metrics is essential in both clinical and research settings.

Echo-planar imaging (EPI)-based DTI is robust against patient motion because of its single-shot acquisition. However, EPI-based DTI is susceptible to distortion due to its sensitivity to static magnetic field (B_0_) inhomogeneity and eddy currents induced by motion-probing gradients (MPG). Such distortions can lead to misinterpretation of tissue microstructure and may affect clinical decision-making. Although post-processing tools such as "topup" and "eddy" [4] in the FMRIB (Functional Magnetic Resonance Imaging of the Brain) Software Library (FSL) are widely used for distortion correction in research, they require offline processing and substantial time, limiting their applicability in routine clinical settings. In particular, these tools often require dedicated computing resources and approximately one to two hours of processing per subject, which can be burdensome in clinical workflows and hinder the broader adoption of advanced DTI metrics in daily practice.

Several studies have investigated diffusion-weighted imaging (DWI) with reverse encoding distortion correction (RDC) [5], which can be obtained directly on the MRI scanner. Ito et al. demonstrated that RDC DWI improves the visualization of nonfunctioning pituitary neuroendocrine tumors compared with conventional DWI [6]. Similarly, Furuta et al. reported that RDC DWI enhances image quality and reduces distortion in DWI of head and neck tumors [7]. Although RDC has been shown to improve image quality in DWI of pituitary and head and neck tumors [6,7], its application to DTI metrics such as FA and ADC has not been extensively validated. DTI with RDC (RDC DTI) acquires images with reversed phase-encoding directions to estimate and correct for echo planar imaging (EPI) distortions directly on the scanner, thereby eliminating the need for separate offline tools. This scanner-based correction approach has the potential to facilitate the clinical adoption of advanced DTI metrics by removing time-consuming offline processing. Building on these findings, RDC DTI has the potential to provide distortion-reduced FA and ADC while substantially reducing post-processing time by bypassing topup and eddy in the FSL. Moreover, RDC DTI may facilitate broader adoption of advanced DTI metrics in routine clinical practice, enabling more reliable assessment of white matter integrity across various neurological and systemic diseases.

This study aimed to visually assess distortions in non-RDC FA (FA_non-RDC_), topup- and eddy-corrected FA in FSL (FA_FSL_), and FA_RDC_ relative to MPRAGE (Magnetization-Prepared Rapid Gradient-Echo), and to perform quantitative validation of FA_RDC_ and ADC_RDC_ for clinical application of RDC DTI.

## Materials and methods

This was a prospective study conducted at Nagoya University Hospital, Nagoya, Aichi, Japan, from August 27, 2024, to April 30, 2025. The study was approved by the Ethics Committee of Nagoya University Hospital (approval number: 2024-0173, dated August 27, 2024). Written informed consent was obtained from all participants.

Study population

Inclusion criteria were healthy adult volunteers aged 18 years or older, who received a full explanation of the study and provided written informed consent, and had no history of neurological disease, neurosurgery, or contraindications to MRI. The exclusion criterion was any report of physical discomfort or other health issues during the examination. A total of healthy volunteers (mean age, 40.1 ± 5.7 years) were finally included in the study.

A sample size of 10 was chosen because previous studies [5] evaluating RDC DWI used a similar number of participants, and our study was designed as a pilot validation study consistent with those reports. All volunteers who fulfilled the inclusion criteria and consented during the study period were included consecutively. 

MRI acquisition

Imaging was performed using a 32-channel coil on a 3 T MRI scanner (Vantage Centurian, version 8.0; Canon Medical Systems, Tochigi, Japan). Acquisitions included b_0_ images without RDC (non-RDC b_0_ images), DTI without RDC (non-RDC DTI), RDC DTI, and MPRAGE.

**Figure 1:**
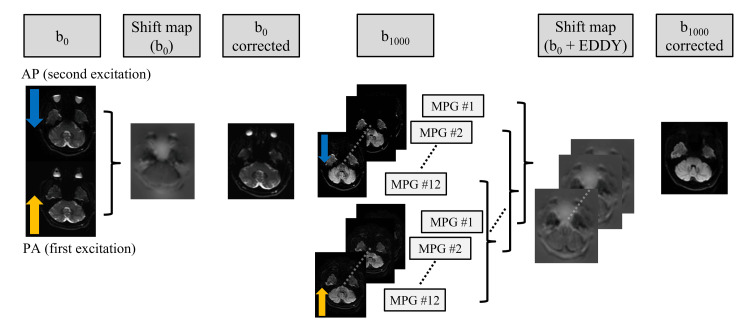
Distortion correction workflow using reverse encoding distortion correction (RDC). AP: anterior–posterior; PA: posterior–anterior; MPG: motion-probing gradient

The distortion correction workflow using RDC is illustrated in Figure 1. Shift maps were generated from b_0_ images acquired with phase-encoding directions in both anterior-posterior (AP) and posterior-anterior (PA) orientations to correct for B_0_ inhomogeneity-derived EPI distortions. These shift maps were applied to the corresponding original b_0_ images, and the corrected images were subsequently averaged to generate the EPI distortion-corrected b_0_ images. Similarly, for each MPG, shift maps (b_0_ + eddy) were generated from images acquired with AP and PA phase-encoding directions. The shift maps were applied to the respective original images, and the corrected images were subsequently averaged to generate EPI- and MPG-derived distortion-corrected images. In this manner, distortion correction using RDC was completely automated, requiring no manual parameter input from the operator.

Imaging planes were aligned parallel to the anterior commissure-posterior commissure line. The common imaging parameters for non-RDC b_0_ images, non-RDC DTI, and RDC DTI were as follows: repetition time (TR), 9000 ms; echo time, 83 ms; field of view, 260 × 260 mm^2^; voxel size, 2 × 2 × 2 mm^3^; number of slices, 66; echo space, 0.7 ms; number of excitations, 2; reduction factor, 3. The phase-encoding direction was AP for non-RDC b_0_ images, PA for non-RDC DTI, and PA (first excitation) and AP (second excitation) for RDC DTI. The specific imaging parameters were: number of MPG directions, 12; b values, 0 and 1000 s/mm^2^; acquisition time, 0:54 for non-RDC b_0_ images, 4:30 for non-RDC DTI, and 4:48 for RDC DTI.

Image analysis

**Figure 2:**
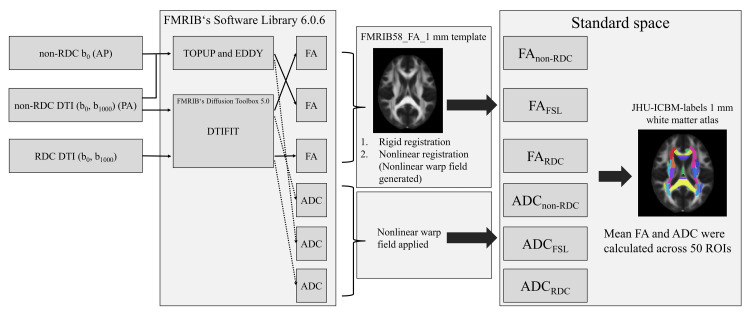
Flowchart of the analysis procedure. DTI: diffusion tensor imaging; RDC: reverse encoding distortion correction; FMRIB: Functional Magnetic Resonance Imaging of the Brain; FA: fractional anisotropy; FNIRT: FMRIB's Non-linear Image Registration Tool; ADC: apparent diffusion coefficient; ROI: regions of interest; JHU-ICBM: Johns Hopkins University-International Consortium of Brain Mapping

Pre-registration FA and ADC were visually assessed to ensure the absence of motion artifacts. The flowchart of the analysis procedure is shown in Figure 2. Images were transferred to a personal computer (AMD Ryzen™ 7 desktop processor, 2 GHz; Advanced Micro Devices, Inc., Santa Clara, California, United States), and all FA and ADC were calculated using the FSL version 6.0.6 on a virtual operating system (VirtualBox, version 7.0; Oracle Corporation, Austin, Texas, United States) configured with four allocated central processing unit (CPU) cores and 8 GB of memory. Distortion correction of non-RDC DTI was performed using non-RDC b_0_ images and non-RDC DTI with topup and eddy in FSL.

All FAs were initially aligned to the FMRIB58_FA_1 mm template using rigid-body registration, followed by nonlinear registration (FMRIB's Non-linear Image Registration Tool (FNIRT)). The corresponding nonlinear warp field was generated as an output of FNIRT. Because no standard template for ADC is available in FSL, ADCs were registered to standard space using the nonlinear warp field obtained from image registration between FA and the template images. Finally, mean FA and ADC values were calculated across 50 regions of interest (ROIs) defined using the Johns Hopkins University-International Consortium of Brain Mapping (JHU-ICBM)-labels 1 mm white matter atlas.

Statistical analysis

Statistical analyses were performed using R software version 4.3.3 (R Foundation for Statistical Computing, Vienna, Austria, https://www.R-project.org/). Bonferroni-corrected Wilcoxon signed-rank tests were applied to compare FA_RDC_ with FA_non-RDC_, FA_RDC_ with FA_FSL_, ADC_RDC_ with ADC_non-RDC_, and ADC_RDC_ with ADC_FSL_. Bonferroni correction was applied separately to FA and ADC. For each metric, multiple comparisons were adjusted independently within each of the 50 ROIs. Statistical significance was defined as P < 0.05.

## Results

The processing time for RDC DTI was approximately two minutes, whereas topup and eddy in FSL required about 90 minutes per subject.

**Figure 3:**
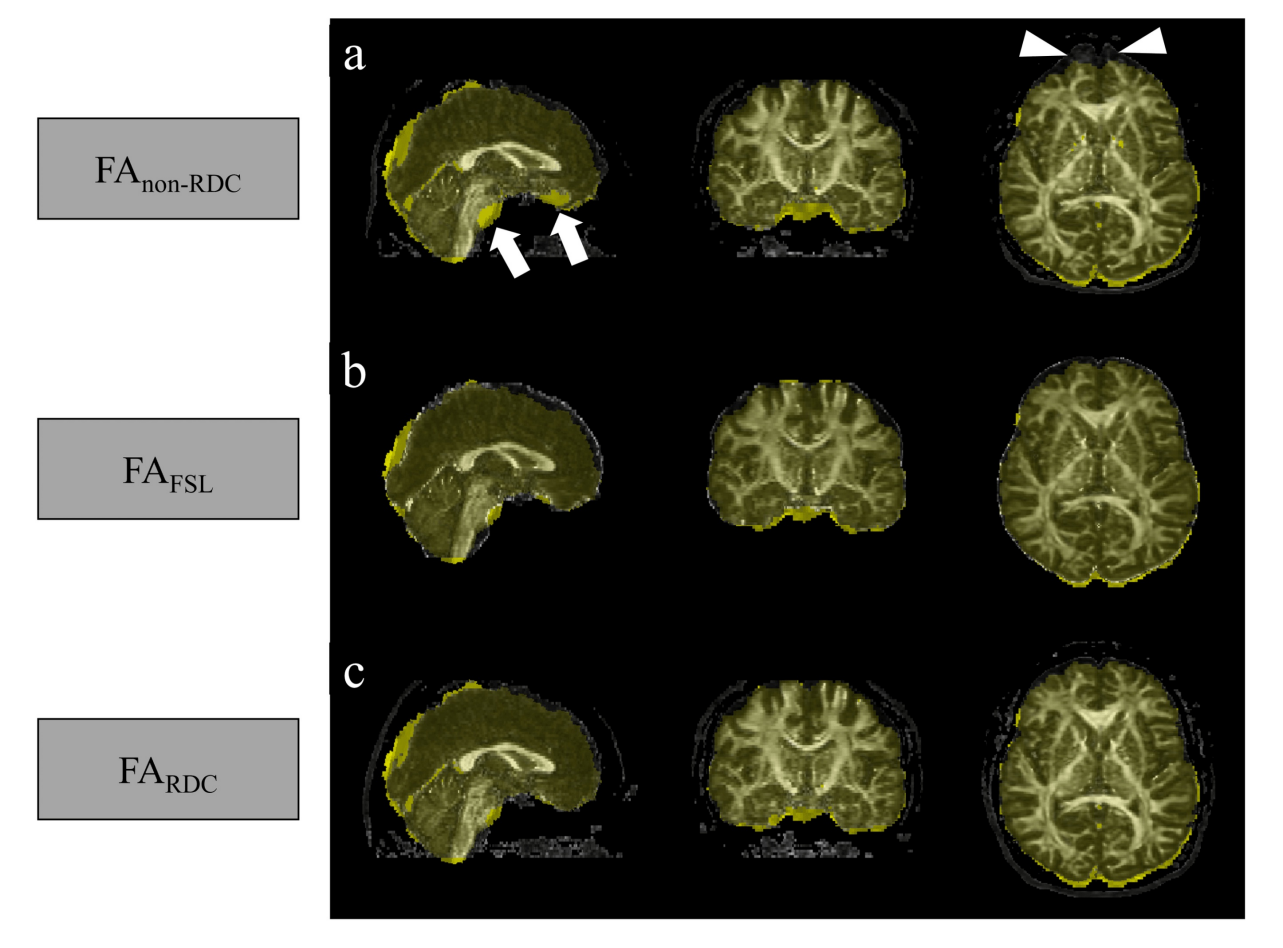
Example of distortion comparison. FA: fractional anisotropy; RDC: reverse encoding distortion correction; FSL: FMRIB (Functional Magnetic Resonance Imaging of the Brain) Software Library

An example comparing distortions is shown in Figure 3. FA_non-RDC_ (Figure 3a), FA_FSL_ (Figure 3b), and FA_RDC_ (Figure 3c) were overlaid with MPRAGE, which is shown in yellow. Distortions observed at the cranial base (arrows) and near the frontal sinuses (arrowheads) on FA_non-RDC_ were substantially mitigated in FA_RDC_, comparable to FA_FSL._

**Figure 4:**
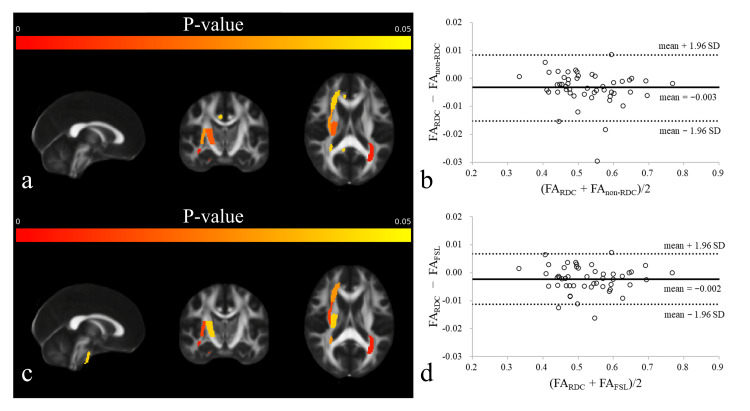
Validation of fractional anisotropy (FA). (a) P-value map for the comparison of FA_non-RDC_ versus FA_RDC_, (b) Bland–Altman plot for the comparison of FA_non-RDC_ versus FA_RDC_, (c) P-value map for the comparison of FA_FSL_ versus FA_RDC_, and (d) Bland–Altman plot for the comparison of FA_FSL_ versus FA_RDC_. Although 10 regions of interest (ROIs) in FA_non-RDC_ and 9 ROIs in FA_FSL_ showed statistically significant differences compared with FA_RDC_, the FA differences for each ROI were minimal, with a mean ± 95% CI of −0.003 ± 0.006 and −0.002 ± 0.005. In the P-value maps (a, c), each voxel is colored according to the statistical significance of FA differences (warmer colors indicate lower P-values). In the Bland–Altman plots (b, d), the horizontal axis shows the mean FA values of the two methods, and the vertical axis shows their difference (FA_RDC_ minus FA_non-RDC_ or FA_FSL_); solid lines indicate the mean difference, and dashed lines show the 95% CI.

**Figure 5:**
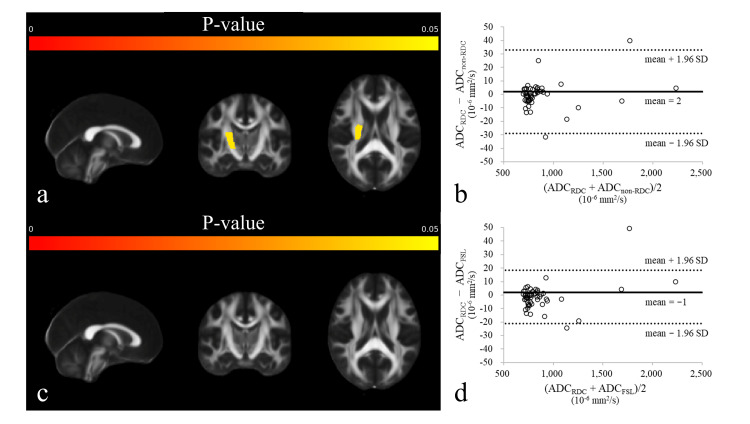
Validation of the apparent diffusion coefficient (ADC). (a) P-value map for the comparison of ADC_non-RDC_ versus ADC_RDC_, (b) Bland–Altman plot for the comparison of ADC_non-RDC_ versus ADC_RDC_, (c) P-value map for the comparison of ADC_FSL_ versus ADC_RDC_, and (d) Bland–Altman plot for the comparison of ADC_FSL_ versus ADC_RDC_. Two regions of interest (ROIs) in ADC_non-RDC_ and none in ADC_FSL_ showed statistically significant differences compared with ADC_RDC_; however, the ADC differences for each ROI were minimal, with a mean ± 95% CI of 2 ± 16 and −1 ± 10 × 10^-6^ mm^2^/s. In the P-value maps (a, c), each voxel is colored according to the statistical significance of ADC differences (warmer colors indicate lower P-values). In the Bland–Altman plots (b, d), the horizontal axis shows the mean ADC values of the two methods, and the vertical axis shows their difference (ADC_RDC_ minus ADC_non-RDC_ or ADC_FSL_); solid lines indicate the mean difference, and dashed lines show the 95% CI.

The mean and standard deviation (SD) of FA and ADC across 50 ROIs are presented in Table 1. Although 10 ROIs in FA_non-RDC_ and nine ROIs in FA_FSL_ showed significant differences compared with FA_RDC_, the FA differences for each ROI were minimal, with a mean ± 95% CI of −0.003 ± 0.006 and −0.002 ± 0.005 (Figure 4). Furthermore, two ROIs in ADC_non-RDC_ and none in ADC_FSL_ showed significant differences compared with ADC_RDC_; however, the ADC differences for each ROI were minimal, with a mean ± 95% CI of 2 ± 16 and −1 ± 10 × 10^-6^ mm^2^/s (Figure 5).

**Table 1:** Summary of FA and ADC values Bonferroni-corrected Wilcoxon signed-rank tests were applied for statistical tests; V-value, test statistics value *, Statistically significant at P < 0.05 FA: fractional anisotropy; ADC: apparent diffusion coefficient; FSL: FMRIB (Functional Magnetic Resonance Imaging of the Brain) software Library; L: left; R: right

ROI		FA			ADC (×10^-6^ mm^2^/s)		
		non-RDC, mean±SD (P-value, V-value)	FSL, mean±SD (P-value, V-value)	RDC, mean±SD	non-RDC, mean±SD (P-value, V-value)	FSL, mean±SD (P-value, V-value)	RDC, mean±SD
1	Middle cerebellar peduncle	0.521 ± 0.012 (0.252, 10)	0.520 ± 0.011* (0.041, 8)	0.515 ± 0.013	938 ± 67 (0.177, 9)	922 ± 45 (0.085, 6)	906 ± 61
2	Pontine crossing tract	0.505 ± 0.043 (0.826, 16)	0.504 ± 0.040 (1.000, 25)	0.493 ± 0.040	740 ± 31 (1.000, 34)	741 ± 25 (1.000, 35)	747 ± 38
3	Genu of corpus callosum	0.601 ± 0.019 (1.000, 22)	0.600 ± 0.019 (1.000, 29)	0.599 ± 0.018	1073 ± 84 (0.222, 46)	1083 ± 83 (1.000, 18)	1080 ± 77
4	Body of corpus callosum	0.651 ± 0.026 (1.000, 27)	0.651 ± 0.026 (1.000, 32)	0.651 ± 0.026	899 ± 45 (1.000, 28)	899 ± 46 (1.000, 27)	900 ± 49
5	Splenium of corpus callosum	0.769 ± 0.009 (0.826, 16)	0.767 ± 0.009 (1.000, 34)	0.767 ± 0.011	771 ± 26 (1.000, 19)	773 ± 26 (0.697, 15)	768 ± 30
6	Fornix (column and body of fornix)	0.442 ± 0.049 (1.000, 31)	0.445 ± 0.050 (1.000, 30)	0.444 ± 0.053	1692 ± 204 (1.000, 26)	1682 ± 210 (1.000, 25)	1687 ± 192
7	Corticospinal tract R	0.568 ± 0.024* (0.006, 0)	0.555 ± 0.026 (0.252, 16)	0.539 ± 0.040	874 ± 93 (0.085, 50)	920 ± 95 (1.000, 36)	933 ± 96
8	Corticospinal tract L	0.586 ± 0.031* (0.012, 1)	0.572 ± 0.031 (1.000, 25)	0.568 ± 0.033	862 ± 83* (0.018, 53)	931 ± 106 (1.000, 28)	928 ± 109
9	Medial lemniscus R	0.626 ± 0.019 (1.000, 25)	0.626 ± 0.021 (1.000, 28)	0.625 ± 0.028	775 ± 41 (1.000, 20)	776 ± 50 (1.000, 22)	762 ± 33
10	Medial lemniscus L	0.632 ± 0.018 (0.480, 13)	0.631 ± 0.020 (0.146, 12)	0.622 ± 0.023	763 ± 31 (1.000, 23)	766 ± 36 (1.000, 19)	758 ± 31
11	Inferior cerebellar peduncle R	0.473 ± 0.041 (1.000, 26)	0.481 ± 0.045 (0.193, 13)	0.473 ± 0.050	838 ± 49 (0.341, 44)	864 ± 68 (1.000, 29)	863 ± 70
12	Inferior cerebellar peduncle L	0.477 ± 0.029 (0.316, 11)	0.482 ± 0.029 (0.252, 15)	0.473 ± 0.035	884 ± 66 (1.000, 30)	895 ± 64 (1.000, 20)	888 ± 88
13	Superior cerebellar peduncle R	0.604 ± 0.022 (0.111, 7)	0.601 ± 0.023 (0.480, 15)	0.599 ± 0.020	1142 ± 75 (0.316, 11)	1148 ± 82 (0.393, 12)	1124 ± 76
14	Superior cerebellar peduncle L	0.576 ± 0.020 (0.697, 15)	0.572 ± 0.020 (1.000, 37)	0.572 ± 0.019	1258 ± 83 (0.662, 15)	1267 ± 85 (0.465, 10)	1248 ± 79
15	Cerebral peduncle R	0.698 ± 0.017 (0.193, 9)	0.694 ± 0.018 (1.000, 21)	0.692 ± 0.013	751 ± 25 (1.000, 19)	754 ± 25 (1.000, 19)	746 ± 24
16	Cerebral peduncle L	0.693 ± 0.017 (1.000, 22)	0.689 ± 0.019 (1.000, 35)	0.692 ± 0.015	743 ± 27 (0.722, 16)	747 ± 33 (0.308, 11)	738 ± 28
17	Anterior limb of internal capsule R	0.572 ± 0.024 (0.826, 16)	0.571 ± 0.022 (1.000, 28)	0.569 ± 0.024	699 ± 40 (1.000, 26)	699 ± 38 (1.000, 21)	699 ± 43
18	Anterior limb of internal capsule L	0.548 ± 0.020 (1.000, 28)	0.548 ± 0.020 (1.000, 33)	0.549 ± 0.023	735 ± 34 (0.057, 4)	736 ± 34 (0.082, 6)	722 ± 42
19	Posterior limb of internal capsule R	0.650 ± 0.016* (0.018, 2)	0.650 ± 0.015* (0.041, 7)	0.646 ± 0.016	703 ± 17* (0.048, 52)	704 ± 16 (0.367, 36)	707 ± 19
20	Posterior limb of internal capsule L	0.645 ± 0.015 (1.000, 21)	0.645 ± 0.014 (1.000, 27)	0.645 ± 0.014	716 ± 19 (0.480, 13)	715 ± 20 (0.658, 15)	712 ± 21
21	Retrolenticular part of internal capsule R	0.594 ± 0.032 (0.059, 5)	0.594 ± 0.031 (0.082, 6)	0.587 ± 0.028	746 ± 33 (1.000, 23)	744 ± 34 (1.000, 22)	744 ± 32
22	Retrolenticular part of internal capsule L	0.590 ± 0.021 (0.111, 48)	0.591 ± 0.021 (0.316, 53)	0.598 ± 0.022	754 ± 24 (0.124, 7)	752 ± 24 (0.606, 15)	745 ± 23
23	Anterior corona radiata R	0.445 ± 0.016* (0.041, 4)	0.444 ± 0.015* (0.029, 4)	0.440 ± 0.014	763 ± 44 (1.000, 30)	763 ± 43 (1.000, 30)	764 ± 42
24	Anterior corona radiata L	0.442 ± 0.019 (0.580, 14)	0.442 ± 0.020 (1.000, 21)	0.440 ± 0.017	777 ± 50 (0.938, 14)	777 ± 50 (1.000, 22)	775 ± 49
25	Superior corona radiata R	0.500 ± 0.021 (0.697, 40)	0.500 ± 0.021 (0.193, 54)	0.501 ± 0.021	714 ± 35 (0.140, 48)	715 ± 35 (0.414, 36)	718 ± 35
26	Superior corona radiata L	0.492 ± 0.022 (0.193, 46)	0.491 ± 0.022 (0.111, 58)	0.495 ± 0.022	733 ± 36 (1.000, 16)	734 ± 36 (1.000, 19)	732 ± 39
27	Posterior corona radiata R	0.495 ± 0.024 (1.000, 36)	0.495 ± 0.024 (0.967, 44)	0.498 ± 0.022	807 ± 44 (1.000, 25)	807 ± 44 (1.000, 26)	808 ± 46
28	Posterior corona radiata L	0.470 ± 0.022 (0.393, 43)	0.469 ± 0.022* (0.041, 57)	0.472 ± 0.020	818 ± 50 (1.000, 33)	817 ± 51 (1.000, 27)	818 ± 52
29	Posterior thalamic radiation R	0.596 ± 0.016 (0.059, 5)	0.596 ± 0.016 (0.082, 8)	0.592 ± 0.016	789 ± 44 (0.506, 42)	790 ± 43 (1.000, 37)	792 ± 47
30	Posterior thalamic radiation L	0.593 ± 0.025* (0.006, 0)	0.592 ± 0.024* (0.006, 1)	0.586 ± 0.025	840 ± 65 (0.377, 43)	841 ± 65 (1.000, 34)	845 ± 73
31	Sagittal stratum R	0.542 ± 0.024* (0.006, 0)	0.540 ± 0.023* (0.006, 0)	0.535 ± 0.022	834 ± 39 (1.000, 36)	834 ± 37 (0.922, 38)	836 ± 33
32	Sagittal stratum L	0.496 ± 0.017 (1.000, 28)	0.494 ± 0.016 (0.193, 54)	0.496 ± 0.015	874 ± 46 (1.000, 29)	876 ± 45 (1.000, 26)	876 ± 43
33	External capsule R	0.419 ± 0.017* (0.029, 3)	0.419 ± 0.017* (0.012, 1)	0.414 ± 0.012	726 ± 39 (0.377, 43)	724 ± 38 (0.057, 51)	730 ± 39
34	External capsule L	0.416 ± 0.016 (1.000, 37)	0.415 ± 0.015 (0.580, 50)	0.418 ± 0.017	747 ± 44 (0.227, 7)	748 ± 45 (0.132, 5)	742 ± 47
35	Cingulum (cingulate gyrus) R	0.492 ± 0.036* (0.041, 4)	0.490 ± 0.035 (0.316, 13)	0.485 ± 0.032	741 ± 24 (1.000, 23)	743 ± 26 (1.000, 12)	741 ± 28
36	Cingulum (cingulate gyrus) L	0.554 ± 0.022 (0.252, 10)	0.554 ± 0.022 (0.146, 13)	0.550 ± 0.023	731 ± 30 (0.369, 9)	729 ± 29 (0.377, 12)	726 ± 32
37	Cingulum (hippocampus) R	0.453 ± 0.032* (0.006, 0)	0.450 ± 0.031* (0.006, 0)	0.438 ± 0.028	821 ± 55 (1.000, 35)	822 ± 59 (1.000, 30)	827 ± 57
38	Cingulum (hippocampus) L	0.413 ± 0.042 (1.000, 21)	0.409 ± 0.040 (1.000, 32)	0.409 ± 0.036	833 ± 62 (1.000, 31)	839 ± 64 (1.000, 25)	837 ± 62
39	Fornix (cres) / Stria terminalis R	0.548 ± 0.025 (0.316, 11)	0.547 ± 0.024 (0.697, 17)	0.543 ± 0.022	937 ± 55 (1.000, 22)	941 ± 55 (1.000, 21)	937 ± 51
40	Fornix (cres) / Stria terminalis L	0.538 ± 0.031 (1.000, 30)	0.537 ± 0.030 (0.697, 44)	0.539 ± 0.025	841 ± 28 (1.000, 37)	845 ± 29 (1.000, 24)	842 ± 23
41	Superior longitudinal fasciculus R	0.472 ± 0.025 (0.967, 17)	0.472 ± 0.025 (1.000, 24)	0.470 ± 0.024	727 ± 30 (1.000, 17)	727 ± 30 (1.000, 23)	727 ± 32
42	Superior longitudinal fasciculus L	0.449 ± 0.014 (0.252, 10)	0.448 ± 0.014 (0.480, 16)	0.446 ± 0.013	727 ± 22 (1.000, 5)	727 ± 22 (0.315, 4)	726 ± 22
43	Superior fronto-occipital fasciculus R	0.466 ± 0.033 (0.697, 15)	0.465 ± 0.031 (1.000, 28)	0.463 ± 0.033	713 ± 49 (1.000, 24)	715 ± 47 (1.000, 25)	714 ± 47
44	Superior fronto-occipital fasciculus L	0.460 ± 0.030 (1.000, 27)	0.459 ± 0.029 (1.000, 41)	0.461 ± 0.030	727 ± 38 (0.227, 7)	727 ± 36 (0.198, 9)	716 ± 44
45	Inferior fronto-occipital fasciculus R	0.469 ± 0.017 (0.393, 12)	0.469 ± 0.017 (0.146, 9)	0.465 ± 0.014	769 ± 28 (0.227, 38)	769 ± 28 (0.248, 45)	773 ± 24
46	Inferior fronto-occipital fasciculus L	0.455 ± 0.013 (1.000, 20)	0.455 ± 0.013 (1.000, 21.5)	0.453 ± 0.013	785 ± 30 (0.277, 11)	785 ± 31 (0.342, 12)	779 ± 32
47	Uncinate fasciculus R	0.528 ± 0.026 (0.697, 15)	0.526 ± 0.025 (1.000, 26)	0.524 ± 0.021	767 ± 33 (1.000, 29)	767 ± 37 (1.000, 27)	767 ± 26
48	Uncinate fasciculus L	0.479 ± 0.017 (0.252, 10)	0.479 ± 0.016 (0.697, 19)	0.474 ± 0.018	787 ± 31 (1.000, 20)	784 ± 31 (1.000, 30)	784 ± 27
49	Tapetum R	0.403 ± 0.021* (0.041, 51)	0.403 ± 0.021* (0.029, 62)	0.409 ± 0.022	1749 ± 99 (0.580, 41)	1740 ± 101 (0.146, 47)	1789 ± 122
50	Tapetum L	0.332 ± 0.019 (1.000, 35)	0.331 ± 0.019 (0.967, 45)	0.333 ± 0.019	2230 ± 185 (1.000, 30)	2224 ± 183 (1.000, 33)	2234 ± 206

## Discussion

We demonstrated that RDC DTI effectively reduced distortions while providing FA and ADC values comparable to those of non-RDC DTI and FSL DTI, with a marked reduction in the overall time required for image acquisition. Although several ROIs in non-RDC DTI and FSL DTI showed statistically significant differences compared with RDC DTI, these differences were minimal. As summarized in Table 2, both FA and ADC values obtained with non-RDC DTI and RDC DTI showed differences that were comparable to or smaller than scan-rescan variability across representative white matter regions [8,9]. These findings suggest that the observed FA and ADC differences are unlikely to have a clinical impact.

**Table 2:** Comparison of non-RDC DTI and RDC DTI with scan–rescan variability of non-RDC DTI ^†^ Bonekamp et al., 2007 [8]; ^††^ Bisdas et al., 2008 [9] n/a: not applicable; RDC: reverse encoding distortion correction; DTI: diffusion tensor imaging; FA: fractional anisotropy; ADC: apparent diffusion coefficient, L: left; R: right

ROI	FA				ADC (10^-6^ mm^2^/s)			
	scan, mean±SD	rescan, mean±SD	non-RDC, mean±SD	RDC, mean±SD	scan, mean±SD	rescan, mean±SD	non-RDC, mean±SD	RDC, mean±SD
Genu of corpus callosum	0.770 ± 0.021^†^	0.758 ± 0.031^†^	0.601 ± 0.019	0.599 ± 0.018	817 ± 36^†^	824 ± 32^†^	1073 ± 84	1080 ± 77
Splenium of corpus callosum	0.823 ± 0.071^††^	0.836 ± 0.069^††^	0.769 ± 0.009	0.767 ± 0.011	n/a			
Corticospinal tract R	0.646 ± 0.067^††^	0.570 ± 0.054^††^	0.568 ± 0.024	0.539 ± 0.040	n/a			
Corticospinal tract L	0.672 ± 0.073^††^	0.649 ± 0.061^††^	0.586 ± 0.031	0.568 ± 0.033	n/a			
Cerebral peduncle R	0.670 ± 0.074^†^	0.694 ± 0.039^†^	0.698 ± 0.017	0.692 ± 0.013	750 ± 51^†^	760 ± 54^†^	751 ± 25	746 ± 24
Cerebral peduncle L			0.693 ± 0.017	0.692 ± 0.015			743 ± 27	738 ± 28
Anterior limb of internal capsule R	0.550 ± 0.024^†^	0.563 ± 0.028^†^	0.572 ± 0.024	0.569 ± 0.024	684 ± 14^†^	685 ± 19^†^	699 ± 40	699 ± 43
Anterior limb of internal capsule L			0.548 ± 0.020	0.549 ± 0.023			735 ± 34	722 ± 42
Posterior limb of internal capsule R	0.550 ± 0.024^†^	0.563 ± 0.028^†^	0.650 ± 0.016	0.646 ± 0.016	662 ± 13^†^	660 ± 18^†^	703 ± 17	707 ± 19
Posterior limb of internal capsule L			0.645 ± 0.015	0.645 ± 0.014			716 ± 19	712 ± 21

Tractography provides detailed visualization of the spatial relationships between tumors or lesions and functionally critical white matter pathways [10], including the corticospinal tract, arcuate fasciculus, and optic radiation. This information can help minimize intraoperative injury and maximize postoperative preservation of neurological function, thereby supporting safer and more effective neurosurgical planning [11]. As demonstrated in our study, RDC DTI, which reduces distortions while maintaining FA values comparable to those obtained with conventional methods, may allow for more precise and reliable tractography. This advantage may be particularly beneficial for preoperative planning, in which accurate tractography of the corticospinal tract, arcuate fasciculus, and optic radiation is critical to avoid postoperative neurological deficits.

Cerebrovascular interventions frequently utilize metallic devices such as clips, coils, and stents, which generate significant susceptibility artifacts that can compromise non-RDC DTI. As previous studies have evaluated distortion-insensitive imaging techniques in this context, RDC DTI may also be promising for assessing susceptibility artifacts [12]. This finding suggests that RDC DTI could be useful not only for preoperative planning but also for postoperative follow-up.

The magnitude of B_0_ inhomogeneity-induced distortion in EPI increases proportionally with magnetic field strength, becoming particularly severe at higher fields such as 7T. Given that RDC DTI effectively mitigates distortions at 3T, this approach may prove especially valuable at 7T and beyond, where traditional correction methods often reach their practical limits. Future validation studies at higher field strengths would help determine whether RDC DTI can maintain its efficiency and accuracy advantages in these more challenging imaging environments.

Unlike FSL-based DTI, RDC DTI requires the acquisition of images with reversed phase-encoding directions not only for the b0 images but also for higher b-values, such as b_1000_ images, which can increase scan time. However, by combining RDC DTI with simultaneous multi-slice excitation [13], the TR can be shortened, thereby potentially reducing scan time.

Limitations

This study has several limitations. The number of participants was relatively small, and the age range was narrow. Previous studies have reported that FA is negatively correlated with age [14,15]. Therefore, the range of FA values across ROIs may have been limited. Because of this restricted cohort, the generalizability of our findings to broader populations may be constrained. Future studies that include a larger and more age-diverse cohort will be important to validate the robustness and clinical applicability of RDC DTI. Further limitations of this study are that scanner variability was not evaluated and that only a single set of scanning parameters was examined. Additionally, the impact of varying distortion-related parameters, such as echo space, on RDC correction accuracy was not assessed and warrants further investigation. Finally, distortion was evaluated visually; although improvements were evident at the cranial base and near the frontal sinuses, a quantitative assessment with statistical analysis would be preferable.

## Conclusions

As an initial validation, RDC DTI effectively reduced distortions while yielding FA and ADC values comparable to those of non-RDC DTI and FSL DTI. Future studies should evaluate its performance in patients with diverse pathological conditions and assess its impact on clinical decision-making and patient outcomes.

The marked reduction in processing time from approximately 90 minutes to two minutes highlights the potential of RDC DTI to support broader clinical use. This improvement may be particularly advantageous in acute settings in which timely decision-making is critical, such as preoperative planning for brain tumor resection or stroke evaluation. In addition, the simplification of post-processing workflows could make RDC DTI more feasible for clinical sites without extensive research infrastructure or specialized expertise.
